# Phosphorylation‐induced lateral rearrangements of thylakoid protein complexes upon light acclimation

**DOI:** 10.1002/pld3.39

**Published:** 2018-02-14

**Authors:** Sanna Rantala, Mikko Tikkanen

**Affiliations:** ^1^ Molecular Plant Biology Department of Biochemistry University of Turku Turku Finland

**Keywords:** *Arabidopsis thaliana*, light‐harvesting complex, phosphorylation, photosystem, protein complex, thylakoid membrane

## Abstract

Understanding the mechanistic basis of balanced excitation energy distribution between photosystem II and photosystem I (PSII and PSI) requires detailed investigation of the thylakoid light‐harvesting system composed of energetically connected LHCII trimers. The exact mechanisms controlling the excitation energy distribution remain elusive, but reversible phosphorylation is known to be one important component. Here, we addressed the role of grana margins in regulation of excitation energy distribution, as these thylakoid domains host all the complexes of photosynthetic light reactions with dynamic response to environmental cues. First, the effect of detergents for the thylakoid membrane connectivity is explained. We show that a specific interaction between the separate LHCII trimers as well as between the LHCII trimers and the PSII and PSI–LHCI complexes is a prerequisite for energetically connected and functional thylakoid membrane. Second, we demonstrate that the optimization of light reactions under changing light conditions takes place in energetically connected LHCII lake and is attained by lateral rearrangements of the PSII–LHCII and PSI–LHCI–LHCII complexes depending especially on the phosphorylation status of the LHCII protein isoform LHCB2.

## Abstract 2

One sentence summary

Reversible phosphorylation of LHCB1 and LHCB2 proteins is demonstrated to optimize the dynamics of the functional unit of the photosynthetic light reactions at grana margins.

## 1 INTRODUCTION

Photosynthetic light reactions, involving two light‐driven photosystems (PSII and PSI), convert solar energy into chemical energy in the thylakoid membrane of chloroplasts. Light energy is harvested by two specialized pigment–protein complex systems: the light‐harvesting complex I (LHCI), collecting excitation energy only for PSI and the light‐harvesting complex II (LHCII), either attached to PSII complexes or functioning as a shared antenna system for both photosystems (Grieco, Suorsa, Jajoo, Tikkanen, & Aro, 2015; Wientjes, van Amerongen, & Croce, 2013). LHCIIs are found as trimers consisting of variable quantities of homologous proteins LHCB1–3 (Jansson, 1994), and the trimers are connected to PSII complexes via LHCB4–6 proteins (Boekema, Van Roon, Van Breemen, & Dekker, 1999; Kouril, Dekker, & Boekema, 2012). S‐LHCII is composed of LHCB1 and LHCB2 and is strongly bound to PSII via LHCB5 protein, whereas M‐LHCII contains LHCB1 and LHCB3 and is moderately attached to PSII via LHCB4 and LHCB6 proteins (Boekema et al., 1999; Kouril et al., 2012). The resulting PSII–LHCII complexes may additionally associate with loosely bound L‐LHCII composed of LHCB1, LHCB2, and LHCB3 (Boekema et al., 1999; Kouril et al., 2012; Rantala, Tikkanen, & Aro, 2017).

In addition to LHCII, the core proteins of PSII (CP43 and CP47) and PSI (PSAA and PSAB) among other smaller subunits bind chlorophyll (chl) molecules and harvest light to the respective photosystems. With the absorbed light energy, PSII transfers electrons from water molecules via plastoquinone (PQ) pool to the cytochrome *b*_*6*_ *f* complex (Cyt *b*_*6*_ *f*), from which the electrons are transported via plastocyanin to PSI. Finally, the second electron excitation in PSI leads to reduction in ferredoxin, which in turn catalyses the conversion of NADP^+^ to NADPH. This linear electron transfer produces proton and electrochemical gradient across the thylakoid membrane, which is utilized by ATP synthase catalyzing the production of ATP that, together with NAPDH, is used as an energy source for chloroplast metabolism.

Chloroplast thylakoid membrane of higher plants is distinctly comprised of appressed grana stacks with high molecular density and nonappressed stroma lamellae with lower protein density (Kirchhoff, Tremmel, Haase, & Kubitscheck, 2004). The photosynthetic protein complexes are unevenly distributed between these two compartments: PSII and LHCII reside mainly in the grana stacks, while the bulky‐structured PSI and ATP synthase are found in the nonappressed regions (Andersson & Anderson, 1980; Daum & Kuhlbrandt, 2011; Dekker & Boekema, 2005; Nevo, Charuvi, Tsabari, & Reich, 2012). On the contrary, the exact localization of Cyt *b*_*6*_ *f* has remained elusive (Kirchhoff, Li, & Puthiyaveetil, 2017; Nevo et al., 2012).

Despite the heterogeneity of the protein complexes, functional linear electron transfer requires sufficient physical proximity between PSII, PQ, and Cyt *b*_*6*_ *f* as well as between Cyt *b*_*6*_ *f*, plastocyanin, and PSI. The outermost parts of grana stacks, the grana margins, enable the connection between the grana‐enriched PSII–LHCII and the stroma lamellae‐enriched PSI–LHCI and ATP synthase and have, for this reason, attracted attention already for decades (Albertsson, 2001; Anderson, 1989; Iwai, Yokono, & Nakano, 2014; Kettunen, Tyystjarvi, & Aro, 1991; Suorsa et al., 2015; van der Weij‐de Wit, Ihalainen, van Grondelle, & Dekker, 2007). Stacking of the thylakoid membrane and consequently the distance between the individual components of light reactions are highly dynamic in response to the changes in light quantity and quality (Kirchhoff, 2013).

The underlying molecular mechanisms guiding the functional interactions of thylakoid protein complexes are not yet fully understood. It is, however, well known that the reversible phosphorylation of PSII core and LHCII proteins plays an important role (Mekala, Suorsa, Rantala, Aro, & Tikkanen, 2015; Puthiyaveetil, van Oort, & Kirchhoff, 2017). LHCII subunits LHCB1 and LHCB2 are phosphorylated by the kinase STATE TRANSITION7 (STN7) (Bellafiore, Barneche, Peltier, & Rochaix, 2005; Bonardi et al., 2005), whereas the phosphatase THYLAKOID‐ASSOCIATED PHOSPHATASE38 (Pribil, Pesaresi, Hertle, Barbato, & Leister, 2010)/PROTEIN PHOSPHATASE1 (TAP38/PPH1) (Shapiguzov et al., 2010) is required for the dephosphorylation of LHCII under increasing light intensity. Phosphorylation of the PSII core proteins D1, D2, CP43, and PSBH, on the other hand, relies on the antagonistic actions of the kinase STATE TRANSITION8 (STN8) (Bonardi et al., 2005; Vainonen, Hansson, & Vener, 2005), the phosphatase PHOTOSYSTEM II CORE PHOSPHATASE (Samol et al., 2012), and in addition to some extent by STN7 (Tikkanen, Nurmi, Suorsa, et al., 2008). STN7 and TAP38/PPH1 are known to be essential for the optimization of excitation energy distribution between PSII and PSI, yet so far, no consensus has been reached about the nature of thylakoid light‐harvesting system or the molecular mechanism of phosphorylation‐dependent regulation.

Genetic tools have enabled to identify the kinases and phosphatases behind the reversible phosphorylation and to investigate their function with knockout mutants. Although even the specific roles of LHCB1 and LHCB2 phosphorylation have been elucidated (Pietrzykowska et al., 2014; Rantala et al., 2017), the actual mechanisms optimizing the excitation energy distribution have remained under debate. Generally, this regulation has been explained with the traditional state transition model relying on migrating LHCII antenna. In this view, PSII and PSI are laterally segregated and the excitation energy distribution between them is balance by shifting LHCII trimer between them. Although phosphorylated LHCII and PSI undoubtedly interact (Wientjes, van Amerongen, et al., 2013), this kind of LHCII represents a very small fraction of the total LHCII (Rantala et al., 2017) and cannot alone be responsible for regulation of excitation energy distribution. Moreover, LHCII is phosphorylated throughout the thylakoid membrane including the PSII‐enriched grana core (Grieco, Tikkanen, Paakkarinen, Kangasjarvi, & Aro, 2012; Tikkanen, Nurmi, Suorsa, et al., 2008), in which the phosphorylated LHCII is confined in PSII–LHCII complexes (Wientjes, Drop, Kouril, Boekema, & Croce, 2013). Indeed, rather than itself migrating to PSI‐enriched stroma lamellae, phosphorylated LHCII seems to attract PSI towards the PSII–LHCII‐enriched grana instead (Tikkanen, Nurmi, Suorsa, et al., 2008).

Here, we aimed to improve the holistic view about the thylakoid membrane with respect to the dynamics and functional interactions of the protein complexes. Our specific goals were (i) to explain how detergents can be used to investigate the thylakoid membrane connectivity and the lateral distribution of thylakoid protein complexes and (ii) to apply this method to examine the light‐ and (de)phosphorylation‐induced rearrangements of the thylakoid protein complexes upon light acclimation.

## 2 MATERIALS AND METHODS

### 2.1 Growth conditions

*Arabidopsis thaliana* WT Columbia and mutant lines *stn7*,* stn8*,* stn7stn8,* and *tap38/pph1* were compared in the experiments. The *stn7* mutant lacks STN7 kinase and consequently the LHCII phosphorylation (Bellafiore et al., 2005), whereas the *stn8* mutant is defective in phosphorylation of PSII core (Bonardi et al., 2005). The *stn7stn8* mutant lacks both kinases and therefore the phosphorylation completely (Bonardi et al., 2005). The *tap38/pph1* mutant, on the other hand, is unable to dephosphorylate LHCII (Pribil et al., 2010). The plants were grown at 23°C and 60% relative humidity under an 8‐hr photoperiod of constant moderate white light (GL, 120 μmol photons m^−2^ s^−1^) with OSRAM PowerStar HQIT 400/D Metal Halide lamps as a light source. For high light (HL) treatment, the plants were illuminated 2 hr with 600 μmol photons m^−2^ s^−1^.

### 2.2 Thylakoid isolation

Thylakoid membranes of 6‐week‐old leaves were isolated 1 hr after the beginning of the daily photoperiod (120 μmol photons m^−2^ s^−1^) and after a 2‐hr HL treatment (600 μmol photons m^−2^ s^−1^). First, the rosettes were ground in grinding buffer (50 mM HEPES–NaOH pH 7.5, 330 mM sorbitol, 5 mM MgCl_2_, 0.05% (w/v) BSA, and 10 mM NaF) and filtered through Miracloth. Chloroplasts were collected by centrifugation at with 3,952 *g* for 7 min at 4°C and ruptured osmotically in shock buffer (50 mM HEPES–NaOH pH 7.5, 5 mM sorbitol, 10 mM MgCl_2_, and 10 mM NaF). The released thylakoid membranes were collected by centrifugation at 3,952 *g* for 7 min at 4°C and suspended in storage buffer (50 mM HEPES–NaOH pH 7.5, 100 mM sorbitol, 10 mM MgCl_2_, and 10 mM NaF). Chlorophyll (chl) concentration (chl *a*+*b*) was determined according to Porra, Thompson, and Kriedemann (1989).

### 2.3 Thylakoid fractionation and blue native gel electrophoresis

Isolated thylakoids (0.5 μg/μl chl) were solubilized either with 1% (w/v) digitonin (DIG, Calbiochem) at vigorous shaking for 8 min or with 1% (w/v) *n*‐dodecyl β‐d‐maltoside (DM; Sigma‐Aldrich) for 5 min on ice. Both detergents were diluted into 25BTH20G buffer (25 mM Bis‐Tris/HCl pH 7.0, 20% (w/v) glycerol, and 0.25 mg/ml Pefabloc) (Järvi, Suorsa, Paakkarinen, & Aro, 2011). The solubilized and insolubilized fractions were separated by centrifugation at 18,620 *g* for 25 min at 4°C and re‐suspended in the 25BTH20G buffer. The DIG‐solubilized and DIG‐insolubilized fractions were further treated with DM. Serva Blue G buffer (100 mM Bis‐Tris/HCl pH 7.0, 0.5 M ACA 30% (w/v) sucrose, and 50 mg/ml Serva Blue G) was added to final volume of 10% (v/v) (Järvi et al., 2011). Final samples containing 2 μg chl were loaded on volume basis and analyzed by large pore blue native gel electrophoresis (lpBN‐PAGE) with acrylamide gradient of 3%–12.5% (Järvi et al., 2011).

### 2.4 Gradual fractionation

Isolated thylakoids (0.5 μg/μl chl) were solubilized at vigorous shaking for 8 min with 0%, 0.25%, 0.5%, 1%, or 2% (w/v) DIG in storage buffer. Soluble grana margins and stroma lamellae were separated from the insoluble grana cores by centrifugation at 18,620 *g* for 25 min at 4°C and re‐suspended in either 2.5 mM HEPES–KOH for the chl determination (Porra et al., 1989) (Figure 3) or into storage buffer for SDS‐PAGE (Figure 5). The chl data were statistically analyzed with one‐way ANOVA and Tukey's test (*p* < .05) using Origin (OriginLab, Northampton, MA, USA).

### 2.5 Denaturating gel electrophoresis and immunoblotting

Thylakoids or DIG‐derived thylakoid fractions were dissolved in sample buffer (138 mM Tris–HCl pH 6.8, 6 M urea, 22.2% (v/v) glycerol, 4.3% (w/v) SDS) with 10% β‐mercaptoethanol (Laemmli, 1970) and separated with SDS‐PAGE. For immunoblotting, proteins were transferred on PVDF membrane (Millipore). From the membrane, phosphorylated threonine residues were recognized with P‐Thr antibody (catalogue number 6949S; New England Biolabs), while proteins D1, PSAB, CYTF, ATPF, LHCB1, LHCB2, P‐LHCB1, P‐LHCB2, and LHCB3 were detected with protein‐specific antibodies produced by Agrisera (catalogue numbers AS10704, AS10695, AS08306, AS10 1604, AS01004, AS01003, AS13 2704, AS13 2705, and AS01002, respectively). In the detection, horseradish peroxidase‐linked secondary antibody (Agrisera) and Amersham ECL Western blotting detection reagents (GE Healthcare) were used. The p‐thr blot was subsequently stained with 0.1% Coomassie Brilliant Blue diluted in 40% methanol and 10% acetic acid.

### 2.6 77 K fluorescence measurements

**Figure 1:**
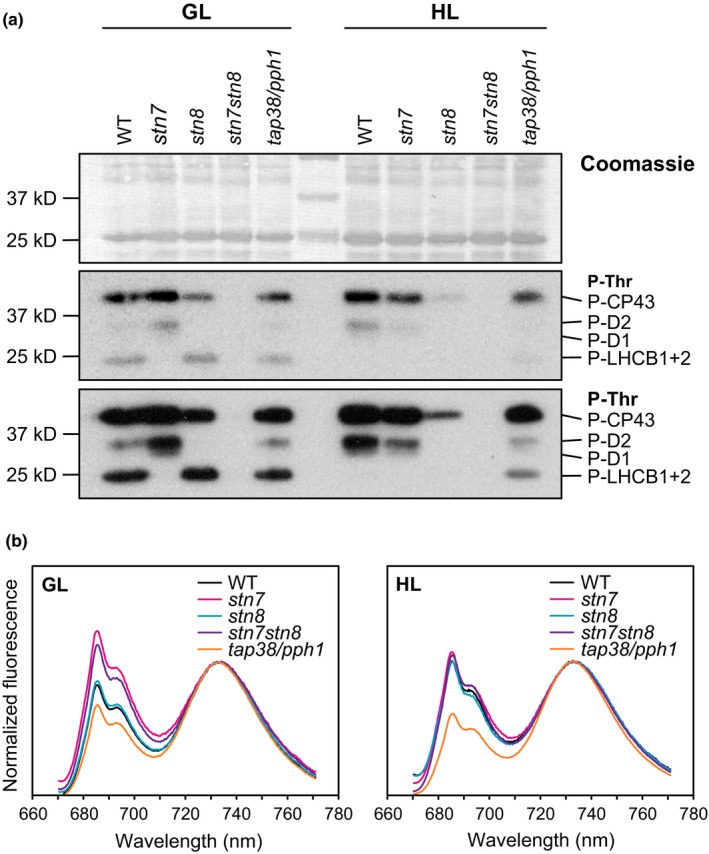
Phosphorylation status of PSII core and LHCII proteins (a) and 77 K fluorescence (b) from WT,* stn7*,* stn8*,* stn7stn8,* and *tap38/pph1* thylakoids. (a) Thylakoids isolated from WT,* stn7*,* stn8*,* stn7stn8,* and *tap38/pph1* plants acclimated to moderate growth light (GL, 120 μmol photons m^−2^ s^−1^) and to high light (HL, 600 μmol photons m^−2^ s^−1^ for 2 hr) were separated in SDS‐PAGE, and phosphorylation levels of CP43, D2, D1, and LHCB1+2 were visualized by immunodetection with P‐Thr‐specific antibody. Two different exposures are shown. Equal loading was confirmed with Coomassie staining. (b) Fluorescence emission from the GL and HL thylakoids was recorded at 77 K. Excitation light of 482.5 nm was used, and the fluorescence spectra were normalized to 733 nm

**Figure 2:**
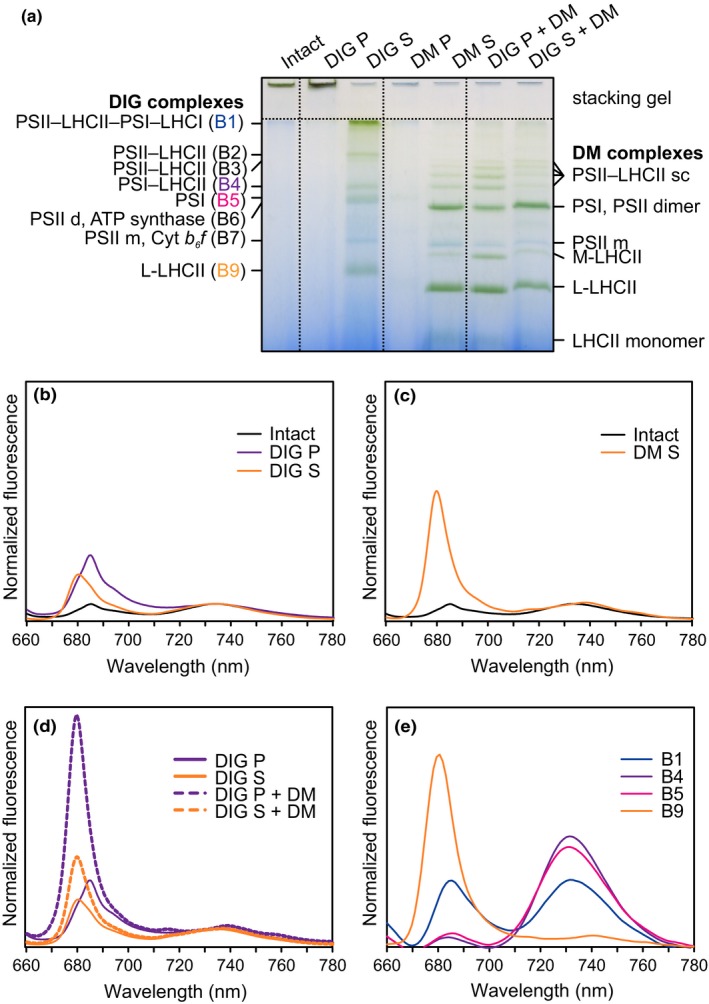
Native protein complexes of differently fractionated WT thylakoid membrane (a) and relative 77 K fluorescence intensity from thylakoids solubilized with detergents (b–e). (a) Thylakoid membranes were isolated from WT plants grown in moderate growth light (120 μmol photons m^−2^ s^−1^) and solubilized with either 1% DIG or 1% DM. The soluble supernatant (S) and the insoluble pellet (P) fractions were then separated, and the S and P from DIG solubilization were further treated with 1% DM. Serva Blue G buffer was added to a final volume of 10% (v/v), and the samples (1 μg of chl *a*+*b*) were analyzed on lpBN‐PAGE containing an acrylamide gradient of 3%–12.5%. (b–d) 77 K fluorescence emission spectra from intact WT thylakoids and from insoluble pellet (P) and soluble supernatant (S) fractions of WT thylakoids after a treatment with DIG (b), DM (c) or both DIG and DM (d). (e). 77 K fluorescence emission spectra from DIG‐soluble protein complexes (B1, B4, B5, and B9) dissected from the lpBN gel (A). For the fluorescence measurements, excitation light of 482.5 nm was used. The data were normalized to 733 nm in (b–d) and to total peak area and in (e)

Fluorescence emission at 77 K was recorded with Ocean Optics S2000 spectrophotometer with 482.5 nm excitation light. For Figure 1b, total thylakoids were diluted to a concentration of 2 μg chl/100 μl with storage buffer, and for Figure 2b–e, DIG‐derived thylakoid fractions were adjusted to a concentration of 2 μg chl/100 μl with 25BTH20G buffer. Data were normalized to 733 nm (Figures 1b and 2b–d) or to the total peak area (Figure 2e).

## 3 RESULTS

### 3.1 Protein phosphorylation in the control of excitation energy distribution

To demonstrate the relationship between protein phosphorylation and the relative excitation of the two photosystems, thylakoids isolated from GL (120 μmol photons m^−2^ s^−1^)‐ and HL (600 μmol photons m^−2^ s^−1^ for 2 hr)‐acclimated WT, *stn7*,* stn8*,* stn7stn8,* and *tap38/pph1* plants were analyzed with respect to P‐Thr antibody signal (Figure 1a) and to relative fluorescence emission spectra at 77 K (Figure 1b). In line with earlier reports, the P‐Thr immunoblot demonstrated the very different phosphorylation state of the lines. WT showed a moderate phosphorylation of both PSII and LHCII in GL, whereas in HL, the phosphorylation of LHCII disappeared and that of PSII strengthened (Figure 1a). In *stn7*, only the PSII core proteins were found phosphorylated, while LHCII phosphorylation was completely absent. In *stn8*, LHCII and CP43 proteins were phosphorylated in GL, whereas in HL, their phosphorylation was decreased. The *stn7stn8* mutant practically lacked all phosphorylation, whereas *tap38/pph1* kept both PSII core and LHCII proteins phosphorylated in both light conditions.

The same samples were subsequently analyzed with respect to their relative excitation of PSII and PSI by recording the fluorescence emission spectra at 77 K (Figure 1b). The spectra normalized to 733 nm revealed distinct differences between the lines. Both *stn7* and *stn7stn8* demonstrated an increased ratio of PSII to PSI excitation as compared to WT, whereas *tap38/pph1* showed an opposite fluorescence emission with reduced PSII to PSI ratio. Despite the very different phosphorylation status (Figure 1a), the fluorescence emission from *stn8* resembled that of WT. After the HL treatment, the differences between WT, *stn7,* and *stn7stn8* disappeared and again *stn8* behaved like WT. Interestingly, however, the lowered ratio of PSII to PSI excitation in *tap38/pph1* was found even pronounced in HL.

### 3.2 Effect of detergents on the functional connections among the light reactions

Although the availability of mutant plants has greatly clarified the physiological role of the thylakoid kinase and phosphatase network (Pesaresi, Pribil, Wunder, & Leister, 2011; Rochaix, 2014), the molecular basis of the phosphorylation‐dependent regulation of excitation energy distribution is still under debate. As the photosynthetic machinery is composed of protein complexes physiologically interacting and eventually resulting in a single connected system, we aimed to elucidate the effect of detergents on the physical interactions between the protein complexes as well as to relative excitation between PSII and PSI. To this end, WT thylakoids were solubilized with (i) 1% digitonin (DIG), (ii) 1% n‐dodecyl β‐D‐maltoside (DM), and (iii) 1% DIG followed by 1% DM. DIG specifically solubilizes the nonappressed membranes leaving the appressed grana core unaffected, while DM is capable of digesting almost the whole thylakoid membrane (Järvi et al., 2011). After the primary (i) DIG and (ii) DM treatments, the insoluble pellet and the soluble supernatant fractions were separated and both subjected to blue native (BN) gel analysis (DIG P, DIG S, DM P, and DM S in Figure 2a). In addition, (iii) both of the insoluble and soluble fractions after DIG were further solubilized with 1% DM and likewise analyzed on BN (DIG P + DM, DIG S + DM in Figure 2a).

Protein complexes of the intact thylakoids as well as of the insoluble fractions from both the DIG‐ and DM‐treated thylakoids remained at the bottom of the gel wells and failed to penetrate the stacking gel (intact, DIG P, and DM P, respectively, in Figure 2a). On the contrary, the soluble fractions after DIG and DM (DIG S and DM S) revealed their characteristic protein complex patterns: DIG S was composed of PSII–LHCII–PSI–LHCI, PSI–LHCI–LHCII, PSI, ATP synthase, PSII monomer + Cyt *b*_*6*_ *f,* and LHCII trimer complexes, whereas DM S consisted of PSII–LHCII supercomplexes, PSI + PSII dimer + ATP synthase, PSII monomer + Cyt *b*_*6*_ *f*, M‐LHCII, L‐LHCII, and LHCII monomer complexes (Järvi et al., 2011).

The insoluble fraction after DIG (DIG P in Figure 2a) was further treated with DM, which enabled the visualization of protein complexes from the appressed membranes that were enriched in PSII–LHCII supercomplexes (DIG P + DM). In addition, the smaller LHCII structures, namely the M‐ and L‐LHCII, were disconnected from the grana‐enriched supercomplexes due to the additional DM solubilization and seen enhanced when compared to the respective bands after one‐step solubilization with DM (DM S). On the contrary, the DM solubilization of the soluble DIG fraction (DIG S + DM) resulted in a similar protein complex pattern as the one‐step DM solubilization (DM S).

Next, 77 K fluorescence emission spectra were recorded from the same thylakoid fractions obtained with DIG, DM, or both (Figure 2b–d, respectively) as well as from separate DIG‐soluble bands B1, B4, B5, and B9 defined in Figure 2a (Figure 2e). Thylakoid fractions solubilized with a detergent, either DIG or DM, resulted not only in the emission peaks at 695 and 730 nm representing PSII and PSI, respectively, but also in a 680‐nm emission peak, which was missing from the intact thylakoids (intact, Figure 2b,c) and from the DIG‐insoluble fractions (DIG P, Figure 2b,d). Comparison of fluorescence emission between the DIG‐soluble and BN‐separated bands B1 (PSII–LHCII–PSI–LHCI), B4 (PSI–LHCII), B5 (PSI), and B9 (L‐LHCII) clearly demonstrated that this fluorescence at 680 nm is mainly emitted from the free L‐LHCII (Figure 2e).

Practically no fluorescence was detected from the insoluble fraction after DM treatment (DM P), whereas fluorescence peak at 685 nm was observed from DIG P (Figure 2b,c, respectively). However, additional DM solubilization of the DIG‐derived thylakoid fractions not only further disassembled the large LHCII‐containing complexes from the nonappressed membranes (DIG S + DM in Figure 2d) but, importantly, also broke the interactions between the grana‐located PSII–LHCII supercomplexes and the LHCII connecting them resulting in L‐LHCII fluorescence at 680 nm (DIG P + DM in Figure 2d). This two‐step solubilization enabled a comparison between the differently appressed membrane fractions, as after DM, both DIG fractions showed a protein complex pattern typical for DM‐solubilized thylakoids, yet with two essential differences (Figure 2a). Firstly, the relative amount of PSII–LHCII supercomplexes, representing the building blocks of highly organized grana thylakoids, was notably higher in the DIG fractions (Figure 2a). Secondly, fluorescence emission from PSI decreased in the DIG‐insoluble fraction and increased in the DIG‐soluble fraction (Figure 2b).

Together the BN and fluorescence data in Figure 2 demonstrate that the DIG‐sensitive fraction is dominated by PSI–LHCI–LHCII complexes and extra LHCII that in the intact system are required to maintain the energetically connected structure of grana margins and stroma lamellae. Further solubilization by DM leads to the disassembly of PSI–LHCI–LHCII complex into PSI–LHCI and LHCII. DIG‐tolerant fraction, in turn, is dominated by PSII–LHCII complexes and the DM‐solubilization results in detachment of LHCII trimers that in the intact system are required to maintain the highly organized and energetically connected structure of grana disks.

### 3.3 Detailed disassembly of GL‐ and HL‐acclimated thylakoid membranes of WT, *stn7*,* stn8*,* stn7stn8,* and *tap38/pph1*

The BN analysis (Figure 2a) indicated that both the solubilization of functional photosynthetic apparatus into protein complexes and the unpacking of larger complexes into their smaller subunits are based on the detachment of LHCII from the thylakoid membrane. To address the role of reversible phosphorylation in the regulation of the individual protein complexes in this energetically connected system, we disassembled the thylakoid membranes of the differently light‐acclimated and phosphorylated WT, *stn7*,* stn8*,* stn7stn8,* and *tap38/pph1* plants with DIG (Grieco et al., 2015).

**Figure 3:**
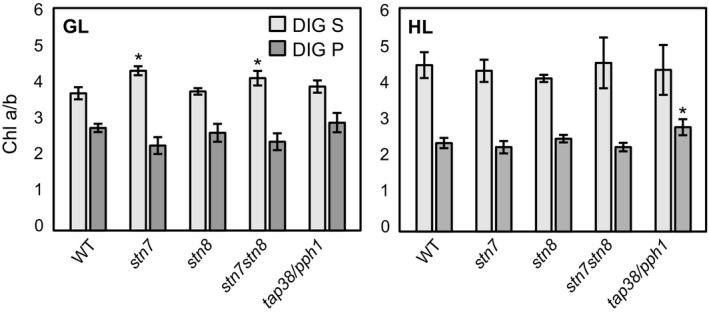
Chl *a*/*b* of the DIG‐soluble and DIG‐insoluble fractions in WT,* stn7*,* stn8*,* stn7stn8,* and *tap38/pph1*. Thylakoid membranes of WT,* stn7*,* stn8*,* stn7stn8,* and *tap38/pph1* plants, harvested from moderate growth light (GL, 120 μmol photons m^−2^ s^−1^) or after high light illumination (HL, 600 μmol photons m^−2^ s^−1^ for 2 hr), were isolated and solubilized with 2% DIG. Chlorophyll *a* to *b* ratio (chl *a*/*b*) was determined from the soluble supernatant (DIG S) and insoluble pellet (DIG P) fractions. Average and standard deviation of three biological replicates are shown, and statistically significant differences between WT and mutants according to one‐way ANOVA and Tukey's test (*p* < .05) are marked with an asterisk (*)

First, to gain an insight of the possible differences in protein complexes detachable with DIG, chl *a* to *b* ratio (chl *a*/*b*) was determined from the soluble and insoluble fraction after 2% DIG (Figure 3). The ratio is an indicator of the relative amount of PSI and LHCII, which are the major contributors to chl *a* and *b* amounts, respectively (van Bezouwen et al., 2017). In GL, *stn7* and *stn7stn8* showed significantly higher values of chl *a*/*b* in soluble supernatant fraction (DIG S) and lower values in insoluble pellet fraction (DIG P) as compared to WT, *stn8,* and *tap38*, which were practically inseparable. As a response to HL treatment (600 μmol photons m^−2^ s^−1^ for 2 hr), the chl *a*/*b* of DIG S rose and that of DIG P decreased in WT and in *stn8* to the levels of *stn7* and *stn7stn8*, whereas in *tap38*, no change was observed, but instead, due to the decrease in WT levels, chl *a*/*b* of the insoluble fraction of *tap38/pph1* showed now a significantly higher value.

**Figure 4:**
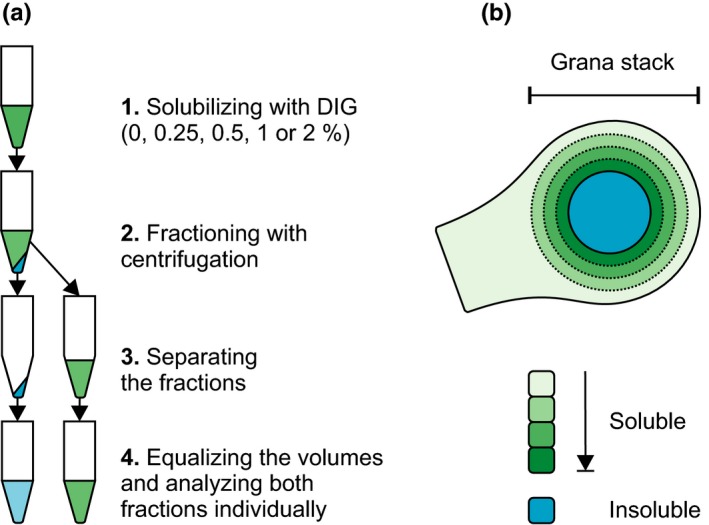
Gradual DIG solubilization and the differently soluble thylakoid membrane. (a) Thylakoid membranes were solubilized with 0%, 0.25%, 0.5%, 1%, and 2% of DIG, after which the soluble fraction (green) was separated from the insoluble fraction (blue) by centrifugation. The two resulting fractions were subsequently individually analyzed. (b) Hypothetical model on the DIG sensitivity of the thylakoid membrane. Small concentrations solubilize only the nonappressed membranes (green), while higher concentrations are capable of breaking stronger interactions between the protein complexes in the nonappressed membranes and especially in the grana margins. The appressed membranes (blue), on the other hand, stay unaffected

**Figure 5:**
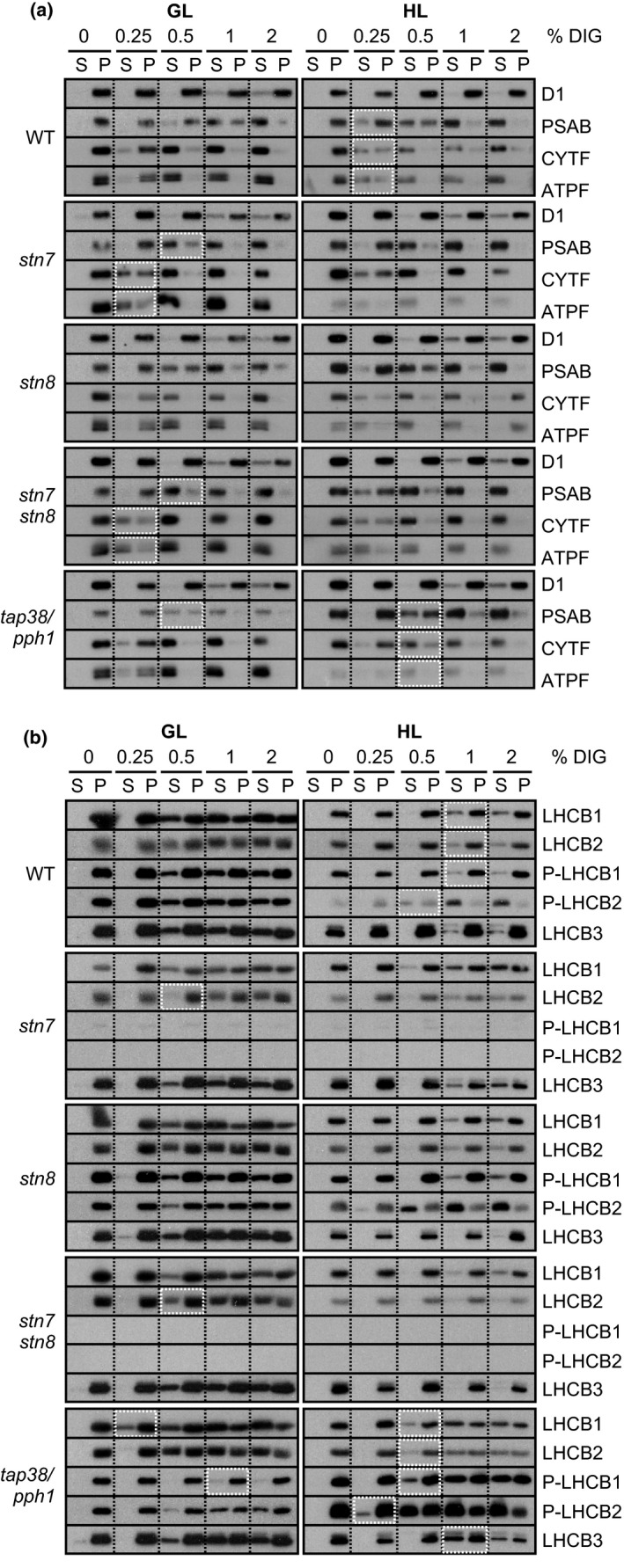
Stepwise detachment of photosynthetic protein complexes from the thylakoid membrane of WT,* stn7*,* stn8*,* stn7stn8,* and *tap38/pph1*. Thylakoid membranes of WT,* stn7*,* stn8*,* stn7stn8,* and *tap38/pph1* plants harvested from moderate growth light (GL, 120 μmol photons m^−2^ s^−1^) or after high light illumination (HL, 600 μmol photons m^−2^ s^−1^ for 2 hr) were isolated and solubilized with 0%, 0.25%, 0.5%, 1%, and 2% DIG. Equal volumes of the soluble supernatant (S) and insoluble pellet (P) fractions were loaded and separated in SDS‐PAGE followed by immunodetection of (a) proteins D1, PSAB, CYTF, and ATPF representing the protein complexes PSII, PSI, LHCII, Cyt *b*_*6*_ *f,* and ATP synthase, respectively, as well as (b) proteins LHCB1, P‐LHCB1, LHCB2, P‐LHCB2, and LHCB3, representing the different subunits of the LHCII complexes. The differences in mutants with respect to WT as well as the differences in WT in response to Hl are marked with white boxes. Representative data from three different biological replicates are shown

The distinct solubilization quality between WT, *stn7*,* stn8*,* stn7stn8,* and *tap38/pph1* thylakoids (Figure 3) suggested differences in membrane structure and/or protein complex interactions according to phosphorylation status of the thylakoid membrane. For this reason, the protein complex composition of DIG‐soluble and DIG‐insoluble fractions was addressed in detail by solubilizing the thylakoid membrane with increasing concentration of DIG (Figure 4) and, subsequently, the detachment of the complexes from each of the DIG‐soluble and DIG‐insoluble fraction was observed (Figure 5). As markers of different electron transport protein complexes, protein‐specific antibodies against D1, PSAB, CYTF, and ATPF were used to represent PSII, PSI, Cyt *b*_*6*_ *f,* and ATP synthase, respectively (Figure 5a), and the localization of different forms of LHCII trimer was determined with antibodies against LHCB1, P‐LHCB1, LHCB2, P‐LHCB2, and LHCB3 (Figure 5b). Antibody signal in the DIG‐soluble supernatant fraction indicated the detachment of the respective protein complex from the thylakoid membrane. In Figure 5, the changes in WT in response to the HL treatment (600 μmol photons m^−2^ s^−1^ for 2 hr) as well as the differences in mutants in respect to WT in the same light condition are marked with white boxes.

Solubilization of the GL‐acclimated WT thylakoids with 0.25% DIG was enough to detach a minor part of CYTF and ATPF into the supernatant fraction, whereas 0.5% was needed to release some of the PSAB and all of the antenna proteins LHCB1, LHCB2, P‐LHCB1, P‐LHCB2, and LHCB3. Higher concentrations, on the other hand, disconnected part of the D1 protein (Figure 5). After the HL treatment, PSAB was released already with 0.25% DIG and, as compared to the GL conditions, CYTF and ATPF were observed in increased amounts in the soluble fraction of 0.25% DIG. LHCB1, LHCB2, P‐LHCB1, and LHCB3 were detached only with 1% DIG in HL, whereas solubilization of P‐LHCB2 remained at 0.5% DIG. Compared to GL conditions, D1 was practically insoluble in HL.

Compared to WT, the *stn7* mutant behaved very differently in GL (Figure 5). As low as 0.25% DIG disconnected over half of the CYTF and the majority of ATPF, whereas 0.5% DIG released already most of the PSAB. No difference in the solubilization of LHCB1 or LHCB3 was noticed, while up to 1% DIG was needed to detach LHCB2. Nevertheless, *stn7* showed no differences to WT in HL.

The *stn8*, mutant behaved like WT, while the detachment pattern on *stn7stn8* resembled that of *stn7* (Figure 5).

In *tap38/pph1*, proteins D1, CYTF, and ATPF expressed similar detachment pattern as in WT, while the detachment of PSAB in GL was slightly enhanced with 0.5% DIG (Figure 5). Disintegration of LHCB2, P‐LHCB2, and LHCB3 resembled that of WT, whereas drastic difference was noticed among the isoforms of LHCB1: the dephosphorylated form of LHCB1 was solubilized already with 0.25% DIG, whereas P‐LHCB1 was practically nonsoluble. Upon the shift to HL, the detachment pattern of *tap38/pph1* differed from all the other lines: in HL, 0.5% DIG was needed to release PSAB, CYTF, and ATPF and, simultaneously, the same concentration was enough to detach the LHCII proteins from the thylakoid membrane of *tap38/pph1*. Noteworthy, P‐LHCB2 was detected in the solubilized fraction already after 0.25% DIG. LHCB3 behaved like the dephosphorylated forms of LHCB1 and LHCB2, found more enriched in the insolubilized fraction.

## 4 DISCUSSION

Investigation of the dynamics of the photosynthetic protein complexes has traditionally included an inherent problem: as demonstrated by the native protein complexes and fluorescence spectra of the thylakoid membrane (Figure 2), a major fraction of the loosely bound L‐LHCII is detached from the system when treating the thylakoid membrane with any detergent. Presumably, this lost LHCII predominantly represents the antenna for PSI, making it problematic to address the PSI light harvesting using detergent‐derived protein complexes. As the fluorescence emission from L‐LHCII is absent from the intact (Figure 2b,c) and mechanically fractionated thylakoids (Tikkanen, Nurmi, Suorsa, et al., 2008), it is evident that detergents destroy the crucial molecular interactions between the light‐harvesting system and the photosystems.

**Figure 6:**
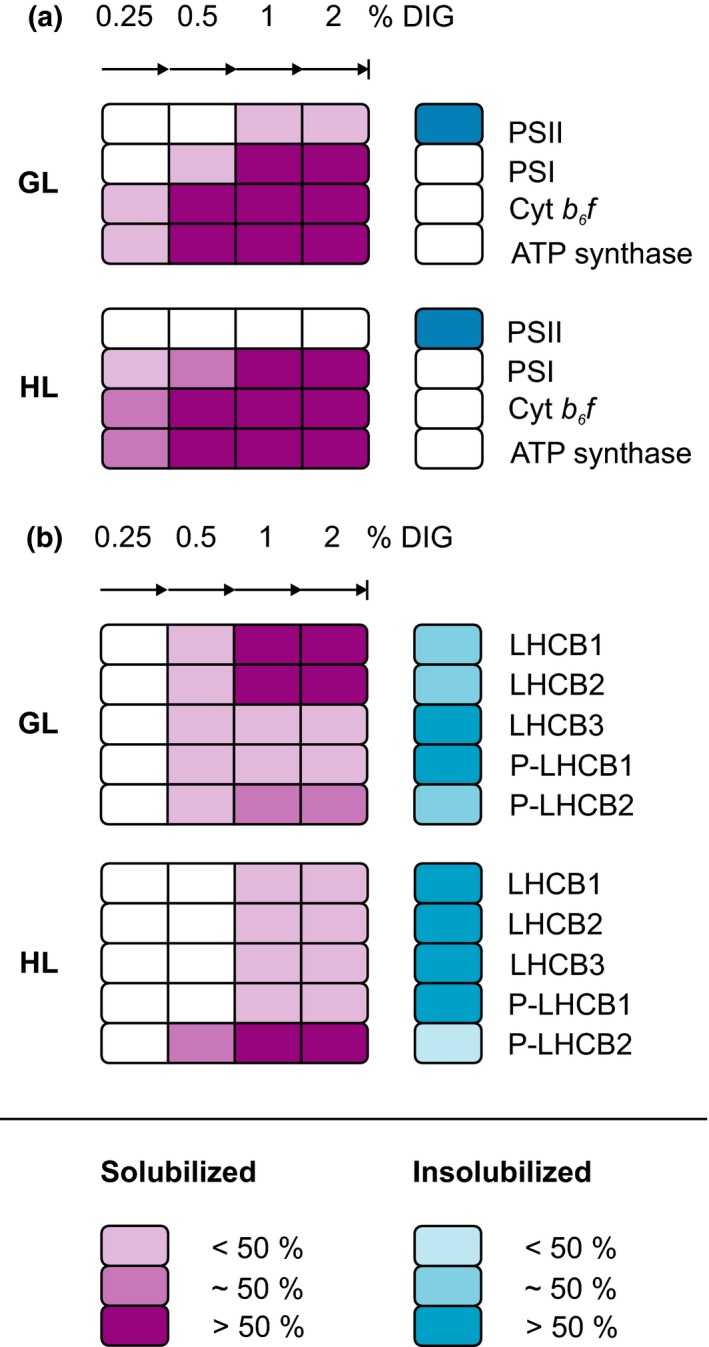
Light‐ and phosphorylation‐dependent rearrangements of the major thylakoid protein complexes. (a) Abundance of PSII, PSI, Cyt *b*_*6*_ *f,* and ATP synthase. In growth light (GL, 120 μmol photons m^−2^ s^−1^) conditions, PSII is mainly found in the insoluble grana core (blue), while PSI, Cyt *b*_*6*_ *f,* and ATP synthase are localized in the highly soluble nonappressed membranes (purple). However, all of these complexes co‐exist in membrane area solubilized with 1% DIG. In response to high light (HL, 600 μmol photons m^−2^ s^−1^ for 2 hr), LHCII phosphorylation vanishes releasing PSI from the contact with LHCII. As a result, PSI, Cyt *b*_*6*_ *f,* and ATP synthase become more susceptible for DIG, while PSII appears insoluble. (b) Abundance of LHCII proteins. In GL, all LHCII proteins are found in the membrane area solubilized with 0.5%–2% DIG as well as in the insoluble grana core. LHCB1 and LHCB2 are especially enriched in the area soluble with 1% and 2% DIG, whereas P‐LHCB1 and LHCB3 are mainly found in the grana core. In HL, LHCII proteins LHCB1, LHCB2, LHCB3, and the slightly phosphorylated form of LHCB1 become tightly packed into DIG‐resistant area, while the opposite is true for phosphorylated LHCB2 following the behavior of PSI, Cyt *b*_*6*_ *f,* and ATP synthase

To overcome the difficulty of studying the PSI light harvesting, it was recently demonstrated that the phosphorylation‐dependent distribution of thylakoid protein complexes between different thylakoids domains can be addressed by gradually solubilizing the membranes with increasing concentration of DIG (Grieco et al., 2015). Using WT and *stn7* mutant plants acclimated to steady‐state growth light, it was shown that LHCII phosphorylation increases the amount of PSI in the thylakoid domain resistant to DIG and enriched in PSII–LHCII complexes (Grieco et al., 2015). Here, we further developed the method and applied it to investigate the effect of light intensity and thylakoid protein phosphorylation on the lateral location of thylakoid protein complexes. For this, we solubilized GL‐acclimated and HL‐treated (600 μmol photons m^−2^ s^−1^ for 2 hr) WT, *stn7*,* stn8*,* stn7stn8,* and *tap38/pph1* thylakoids with five different DIG‐concentrations and, importantly, analyzed both soluble and insoluble fractions (Figure 4a). As illustrated in Figure 4b, solubilization efficiency of DIG depends on its concentration: (i) Low concentration (0.25%) detaches only separate complexes from the nonappressed stroma lamellae, (ii) while higher concentrations (0.5%–2%) are capable of gradually solubilizing the grana margins, (ii) leaving the grana core intact. By immunoblotting each solubilized and insolubilized membrane fraction with protein‐specific antibodies, we could observe the relative abundancies of the protein complexes in the differently soluble areas of the thylakoid membrane (Figures 5 and 6). Based on the proteins detached, it is logical to conclude that the increasing concentration of DIG gradually digests the grana margins and thereby allows a detailed investigation of the rearrangements of the complexes in this area containing a large amount of LHCII trimers and all the components of the electron transport chain (Figures 5 and 6).

The lowest concentration of DIG (0.25%) left the pigment–protein complexes PSII and PSI insoluble, yet was enough to disintegrate part of ATP synthase from the thylakoid membrane (Figure 6a). ATP synthase locates in the stroma lamellae, which as a single membrane layer has no structural protection against DIG and which additionally contains low protein‐to‐lipid ratio further promoting the effect of detergent. Surprisingly, also Cyt *b*_*6*_ *f* was partially detached from the membrane with 0.25%, which is problematic, since as an essential part of the electron transport chain between the photosystems, it is unreasonable to imagine the complex to locate at the stroma lamellae together with ATP synthase. Instead, it is more likely that Cyt *b*_*6*_ *f* stays close to the photosystems but only lacks DIG‐resistant protein–protein interactions with them. In fact, Cyt *b*_*6*_ *f* was recently found to remain separate from the functional megacomplex of light reactions (Rantala et al., 2017), and based on these observations, we suggest that Cyt *b*_*6*_ *f* is embedded in special lipid‐enriched membrane domain easily accessed by DIG. Existence of such hypothetical domains may also be required to accommodate a sufficient amount of PQ in the thylakoid membrane. Concerning the lateral localization of Cyt *b*_*6*_ *f*, it is important to note that the complex was completely absent from the DIG‐resistant thylakoid domain (Figure 6a) without a doubt indicating a location outside the grana core. This contradicts with the previous reports localizing Cyt *b*_*6*_ *f* also in the grana stacks (Johnson, Vasilev, Olsen, & Hunter, 2014; Tikkanen, Nurmi, Suorsa, et al., 2008). Although our results agree with the co‐localization of PSII and Cyt *b*_*6*_ *f* (Johnson et al., 2014), they clearly indicate that this is possible solely outside the tightly appressed grana core.

In contrast to Cyt *b*_*6*_ *f* and ATP synthase, detachment of PSI from the thylakoid membrane required a slightly higher concentration of DIG. In GL‐acclimated plants, PSI was found solubilized to some extent with 0.5% DIG, while the concentrations 1 and 2% released the majority of the complex (Figure 6a). In response to HL, however, PSI was disintegrated already with 0.25% DIG, which can be explained by the light‐induced changes in LHCII phosphorylation (Figure 1a). Losing the phosphorylation as an attractive force, PSI–LHCI drifts apart from PSII–LHCII, seen as decreased relative excitation, increased chl *a*/*b* and increased DIG sensitivity of PSI (Figures 1b, 3, and 6a). In the absence of LHCII phosphorylation (*stn7* and *stn7stn8*), these consequences are present already in GL and, for this reason, no further changes are observed as a response to HL. On the contrary, the constitutively phosphorylated LHCII (*tap38/pph1*) confines PSI to PSII–LHCII, channeling the excitation energy towards PSI and simultaneously protecting PSI from DIG (Figures 2b, 3, and 6a). It is important to note that also the DIG‐resistant fraction contained a substantial amount of PSI strongly suggesting that part of the PSI locates in PSII–LHCII‐enriched thylakoid fraction. As up to three LHCII trimers can be attached to PSI (Bell, Frankel, & Bricker, 2015), our data support the hypothesis that PSI resides in the same energetically connected antenna lake together with PSII (Grieco et al., 2015).

While the role of STN7 decreases in HL, the simultaneously activated STN8 facilitates the unpacking of PSII supercomplexes and dimers in high light (Fristedt et al., 2009; Goral et al., 2010; Tikkanen, Nurmi, Kangasjarvi, & Aro, 2008) and has, for this reason, been proposed to regulate the lateral migration of protein complexes together with STN7‐dependent phosphorylation (Mekala et al., 2015; Tikkanen, Grieco, Kangasjarvi, & Aro, 2010). Interestingly, however, the *stn8* mutant shows no differences from WT in respect to the relative excitation energy or to the distribution of thylakoid protein complexes (Figures 2b, 3, and 5). The reason for these intuitively opposite results may derive from the different viewpoints: the previous studies have focused on the properties of grana membranes and on the sensitivity of PSII complexes for DM (Fristedt et al., 2009; Goral et al., 2010; Tikkanen, Nurmi, Kangasjarvi, et al., 2008). It is nevertheless likely that STN8‐dependent phosphorylation of PSII core proteins affects only the forces inside PSII–LHCII supercomplexes in grana membranes and therefore has no effect on the protein composition of grana margins determining the relative excitation of PSII and PSI. It seems in fact that the LHCII‐mediated excitation of PSI is regulated solely by STN7‐dependent phosphorylation of LHCB1 and LHCB2 proteins and possibly also CP43 (Figures 2b, 3, and 5).

The LHCIIs that can serve light harvesting for both photosystems are trimers built of variable amounts of LHCB1, LHCB2, and LHCB3 proteins (Boekema et al., 1999). To elucidate the distinct roles of LHCB1 and LHCB2 phosphorylation in the lateral distribution of thylakoid protein complexes, we utilized specific antibodies against LHCB1–3 as well as against the phosphorylated forms of LHCB1 and LHCB2 (Pietrzykowska et al., 2014). The extensive immunoblotting analysis of the three LHCII isoforms indicated that, in GL, the dephosphorylated forms of LHCB1 and LHCB2 are in some extent detachable with 0.5%–2% DIG but concentrated in thylakoid area soluble only with 1% and 2% DIG (Figure 6b). The phosphorylated P‐LHCB2 is as well found throughout the thylakoid membrane, while P‐LHCB1 appears enriched in the grana core together with LHCB3 (Figure 6b). As a response to HL, the STN7 kinase is inhibited (Rintamäki, Martinsuo, Pursiheimo, & Aro, 2000) and TAP38/PPH1 allowed to dephosphorylate LHCB1 and LHCB2 (Figure 1a). Nevertheless, P‐LHCB1 still remains partly phosphorylated and enriched in the DIG‐resistant membranes together with LHCB3, but notably, also the dephosphorylated forms are now packed into the grana core (Figure 6b).

In contrast to all the other LHCII subunits, P‐LHCB2 is found remaining in the DIG‐accessible membrane fraction even in HL (Figure 6b). Comparison of the kinase and phosphatase mutants clarified that, indeed, from all the three LHCII isoforms, phosphorylation has a direct effect only on location of LHCB2‐containing L‐LHCII trimers, found enriched in the grana core when dephosphorylated (*stn7* and *stn7stn8*) and, on the contrary, in the nonappressed membranes when highly phosphorylated (*tap38/pph1* in HL) (Figure 5b). Furthermore, the location of P‐LHCB2 followed that of PSI: PSI was more susceptible to DIG in the absence of P‐LHCB2 and, vice versa, well protected when co‐existing with P‐LHCB2 (Figures 5b and 6b). As P‐LHCB2 exclusively exists in the loosely bound L‐LHCII (Rantala et al., 2017) functioning as an efficient antenna for PSI (Wientjes, van Amerongen, et al., 2013), it seems evident that the phosphorylation of LHCB2 increases the physical interaction of PSI in the protein complex network making it more difficult for DIG to detach. Although nearly identical in their amino acid sequence, LHCB1 and LHCB2 have been reported to show very distinct functions (Pietrzykowska et al., 2014). Indeed, our data clearly indicate that phosphorylation of LHCB2 regulates the connection between PSII‐LHCII, L‐LHCII and PSI–LHCI, whereas phosphorylation of LHCB1 seems to be involved in the interaction of separate PSII–LHCII complexes.

## 5 CONCLUSIONS

Taken together, our data collectively demonstrate that the interaction between PSII–LHCII and PSI–LHCI, and consequently, the whole light reaction connectivity is strengthened by STN7‐dependent phosphorylation of LHCII in GL. In HL, this interaction is lost and DIG is able to detach relatively more PSI from the nonappressed membranes, while the majority of LHCII is confined in the grana core with PSII, thus being less accessible to DIG.

## AUTHOR CONTRIBUTIONS

M.T. conceived the original research plan and wrote the article; S.R. performed the experiments and wrote the article.
