# Supplementary material for: Phosphorylation‐induced lateral rearrangements of thylakoid protein complexes upon light acclimation
Source: Plant Direct. 2018 Feb 14;2(2):e00039. doi: 10.1002/pld3.39 (PMC6508491; doi:10.1002/pld3.39)
Supplement: Supplementary file 1 [file PLD3-2-e00039-s001.pdf]

Dear Editors,

We would like to thank the editors and reviewers for the useful comments and suggestions. Both reviewers pointed out the problematic interpretation of Figure 1A. This is now replaced with a new blot and two different exposures to make it easier to distinguish between phosphorylation of PSII core and LHCII. Another main concern was the length and complicatedness of the Discussion. We agree and have now carefully reorganized and restructured the manuscript according to the suggestions. We believe that this improved and clarified the manuscript substantially. Please see below for our more detailed answers to reviewer's comments.

Sincerely,

Mikko Tikkanen

REVIEWER #1

The manuscript Rentla & Tikkanen elucidated the role of pigment-binding protein phosphorylation in thylakoid membrane. The authors focused on physical binding strength among LHCIIs and photosystems. To differentiate the connectivity among the photosynthetic protein complexes, the authors performed stepwise detergent (Digitonine) treatment of thylakoid membranes, which were isolated under growth light (GL) or high-light (HL). As shown in figure 5 and 6, the accessibility of digitonine to the protein complexes in the thylakoid membranes were varied in phosphorylation-dependent manner. Thus, the authors semi-quantitatively determined the physical connectivity among the protein complexes in the isolated thylakoids.

Major comments for the current manuscript

1. The methods used in the study are acceptable except the absence of details of materials. The authors should describe the details of antibodies used for immunoblotting analysis. Although figure 1A only shows "P-LHCII", the figure 5B shows both "P-LHCB1" and "P-LHCB2", arising a question how the author distinguished the difference between "P-LHCB1" and "P-LHCB2". Please provide information about all antibodies; Company, recognizing peptides, etc.

- In the Figure 1A, all phosphorylated threonine residues were detected with an antibody from New England Biolabs, whereas in the Figure 5B, specific antibodies were used to detect P-LHCB1 and P-LHCB2. The reviewer 1 is right, we should have included the antibody information in Materials and methods. The catalogue numbers for the antibodies are now

added but, according to Joanna Porankiewicz-Asplund from Agrisera, the exact protein sequences cannot be provided to protect intellectual property and avoid copying.

2. In general, blotting efficiency, antibody treatment time, and exposure time of immunoblotting analysis cannot be same among different blot images. The authors described "Upon increase in light intensity, ~~~ dephosphorylate LHCII (Figure 1A)" in LINE 432-434 and "As a response to HL, ~~~LHCB1 and LHCB2 (Figure 1A)" in LINE484-486. To quantitatively compare the phosphorylation levels of protein complexes as the authors mentioned, the figure 1A should be shown as a single blot image. Also, immunoblot data of the loading control protein (or CBB stained gel) is suggested to be shown.

- The Figure 1A has been replaced with two different exposures of a new and clearer blot. The upper exposure makes it possible to observe the phosphorylation of PSII core proteins CP43, D2 and D1, while the one below contrasts the phosphorylation of LHCII proteins LHCB1 and LHCB2. In addition, Coomassie staining is now shown to ensure equal loading.

Minor comments

1. Figure 1A shows 5 different bands, while only 4 labels are shown. Readers may be not able to distinguish "which band is which". Please indicate more clearly about those 5 bands.

- Phosphorylated CP43, D2, D1 and LHCB1+2 are now shown as separate labels. P-LHCB1 and P-LHCB2 are of similar size and cannot be distinguished on P-Thr blot and are thus together referred as to P-LHCB1+2.

2. Please unify the mutant name in both manuscript and figures (unify to tap38 or tap38/pph1).

- Name of the mutant is unified to *tap38/pph1*.

3. Please unify the character format of Cytb6f (Italic format).

- Cyt b_6f is now unified to Cyt $\mathit{b_6f}$ (italic).

4. The numbering of figures is strange. Figure 5 appears before Figure 4 in the current manuscript.

- Thank you for noticing! This mistake is corrected by introducing the solubilization method (Figure 4) just before explaining the results of it (Figure 5) in the Results section.

5. LINE 400 "ETC" is described without abbreviation information (probably electron transfer chain?).

- This is true and since the abbreviation was only used two times, it has now been replaced with “electron transport chain”.

6. LINE 452 "Figure 3 and Figure 4" should be "Figure 5 and Figure 6"?

- Yes, they should have been Figure 5 and 6 and are now corrected.

REVIEWER #2

State transitions are mechanisms to regulate the distribution of excitation pressure between two photosystems. But the exact molecular mechanism for the regulation is unclear. In this study, protein complexes were solubilized using two detergents, DIG and DM from WT plants exposed to GL and HL. The same analysis was performed using stn7, stn8, stn7 stn8, and tap38 mutants. They propose a model, in which the phosphorylation of LHCII regulates the lateral rearrangement of PSII-LHCII and PSI-LHCI-LHCII.

The topic of research is very important and the conclusion and discussion are scientifically sound. But I feel some problems in the presentation in this manuscript. The manuscript can become more easily accessible for readers by rewriting the text.

Specific problems.

1) The results section is rather descriptive and I could not understand what could be scientifically concluded from the results. I had to read the long discussion to understand the scientific message of this manuscript. For examples, lines 342-345, 350-369 and 371-374 should be moved to the result section. It may be a good idea to combine results and discussion.

- The parts mentioned are either moved to the Results or deleted.

2) The long discussion also includes the information, which should be explained in introduction (ex. the first two paragraphs). The authors should eliminate any redundancy.

- The long Discussion has now been shortened by compressing the expressions and by either deleting or moving parts of it to the other sections. This was certainly needed since now the Discussion is three pages shorter and the main message clearer.

3) Figure 1A and lines 138-142. The explanation is consistent with the many published results and I do not doubt this statement. But the presented results are very ambiguous. For example, the main

different of the WT patterns between two light conditions observed the second unlabeled band, but the other bands look similar between two conditions. Is it possible to substitute the clearer pattern? Alternatively, the result is dispensable.

- The Figure 1A has been replaced with two different exposures of a new and clearer blot. The upper exposure makes it possible to observe the phosphorylation of PSII core proteins CP43, D2 and D1, while the one below contrasts the phosphorylation of LHCII proteins LHCB1 and LHCB2. In addition, Coomassie staining is now shown to ensure equal loading.

4) Figure 3. Again, the difference is too minor to follow the description in the text. At least, the statistical analysis should be done.

- The chl data was statistically analyzed with one-way Anova after first confirming the normality of the data with Shapiro-Wilk's test. The significant differences according to Tukey's test ($p<0.05$) are now shown with an asterisk.

5) Line 190. I could not follow the reason why M-LHCII was detectable only after the two-step solubilization.

- A fraction of M-trimer is disintegrated also after one-step DM solubilization because DM has a stronger capacity to dissect the protein complexes as compared to digitonin, which maintain larger complexes (see Caffarri et al. 2009, EMBO J.).

6) Figure 4A should be cited before Figure 5.

- Thank you, the order has been corrected.

7) Line 293. As demonstrated in Figure 1A, ... I do not think so.

- This comment was well-founded since, indeed, Figure 1A does not show LHCB1 or LHCB2 but the whole LHCII. This is corrected in the text.
